# Transient ultrasound stimulation has lasting effects on neuronal excitability

**DOI:** 10.1016/j.brs.2021.01.003

**Published:** 2021

**Authors:** Benjamin Clennell, Tom G.J. Steward, Meg Elley, Eunju Shin, Miles Weston, Bruce W. Drinkwater, Daniel J. Whitcomb

**Affiliations:** a Bristol Medical School, Faculty of Health Sciences, University of Bristol, Bristol, BS1 3NY, UK; b Neuroscience and Mental Health Research Institute, Cardiff University, Cardiff, CF24 4HQ, UK; c TWI Technology Centre, Port Talbot, SA13 1SB, UK; d Faculty of Engineering, University of Bristol, Bristol, BS8 1TR, UK

**Keywords:** Ultrasound stimulation, Neuromodulation, Intrinsic excitability, Primary neuron, Whole-cell patch clamp, Electron microscopy

## Abstract

**Background:**

Transcranial ultrasound stimulation can acutely modulate brain activity, but the lasting effects on neurons are unknown.

**Objective:**

To assess the excitability profile of neurons in the hours following transient ultrasound stimulation.

**Methods:**

Primary rat cortical neurons were stimulated with a 40 s, 200 kHz pulsed ultrasound stimulation or sham-stimulation. Intrinsic firing properties were investigated through whole-cell patch-clamp recording by evoking action potentials in response to somatic current injection. Recordings were taken at set timepoints following ultrasound stimulation: 0–2 h, 6–8 h, 12–14 h and 24–26 h. Transmission electron microscopy was used to assess synaptic ultrastructure at the same timepoints.

**Results:**

In the 0–2 h window, neurons stimulated with ultrasound displayed an increase in the mean frequency of evoked action potentials of 32% above control cell levels (*p* = 0.023). After 4–6 h this increase was measured as 44% (*p* = 0.0043). By 12–14 h this effect was eliminated and remained absent 24–26 h post-stimulation. These changes to action potential firing occurred in conjunction with statistically significant differences between control and ultrasound-stimulated neurons in action potential half-width, depolarisation rate, and repolarisation rate, that were similarly eliminated by 24 h following stimulation. These effects occurred in the absence of alterations to intrinsic membrane properties or synaptic ultrastructure.

**Conclusion:**

We report that stimulating neurons with 40 s of ultrasound enhances their excitability for up to 8 h in conjunction with modifications to action potential kinetics. This occurs in the absence of major ultrastructural change or modification of intrinsic membrane properties. These results can inform the application of transcranial ultrasound in experimental and therapeutic settings.

## Highlights

• 40 s of ultrasound stimulation enhances intrinsic excitability of cultured rat neurons.
• Enhanced excitability lasts for up to 8 h.
• Ultrasound has no effects on neuronal ultrastructure.

## 1 Introduction

Exogenous stimulation of the brain has been extensively used to define cognitive processes and map out neural circuitry [1]. Many tools have been developed for this purpose, including implantable electrodes [2] and optogenetics [3], as well as transcranial magnetic stimulation [4] (TMS) and direct current stimulation [5] (tDCS). However, these approaches are inherently limited either by their invasiveness or lack of spatial precision. An emerging approach in this regard is transcranial ultrasound stimulation [6]. Here, a growing number of studies report non-invasive modulation of neural activity of various cortical and subcortical brain regions with millimetre-scale precision [[7], [8], [9], [10]]. This has led to the recent application of the approach in cognitive mapping [9] and as a therapeutic intervention in diseases including Alzheimer’s disease, epilepsy and depression [11].

At the cellular level, ultrasound stimulation can elicit action potential firing [12,13], modulate voltage-gated ion-channel currents [13], and stimulate synaptic transmission [12]. Studies have variously implicated mechanosensitive channels[14], pore-formation[15], membrane cavitation[16] and glial cell activation [17] as key cellular transducers of ultrasound. However, the downstream consequences for neuronal function remain to be fully explored. Specifically, the temporal characteristics of the neuromodulatory effects of ultrasound, including the extent to which they are sustained following stimulation, are currently unknown. To address this, we investigated the excitability profile of neurons in the hours following transient stimulation. Our findings reveal ultrasound can induce sustained modulation of intrinsic neuronal excitability.

## 2 Results

**Fig. 1:**
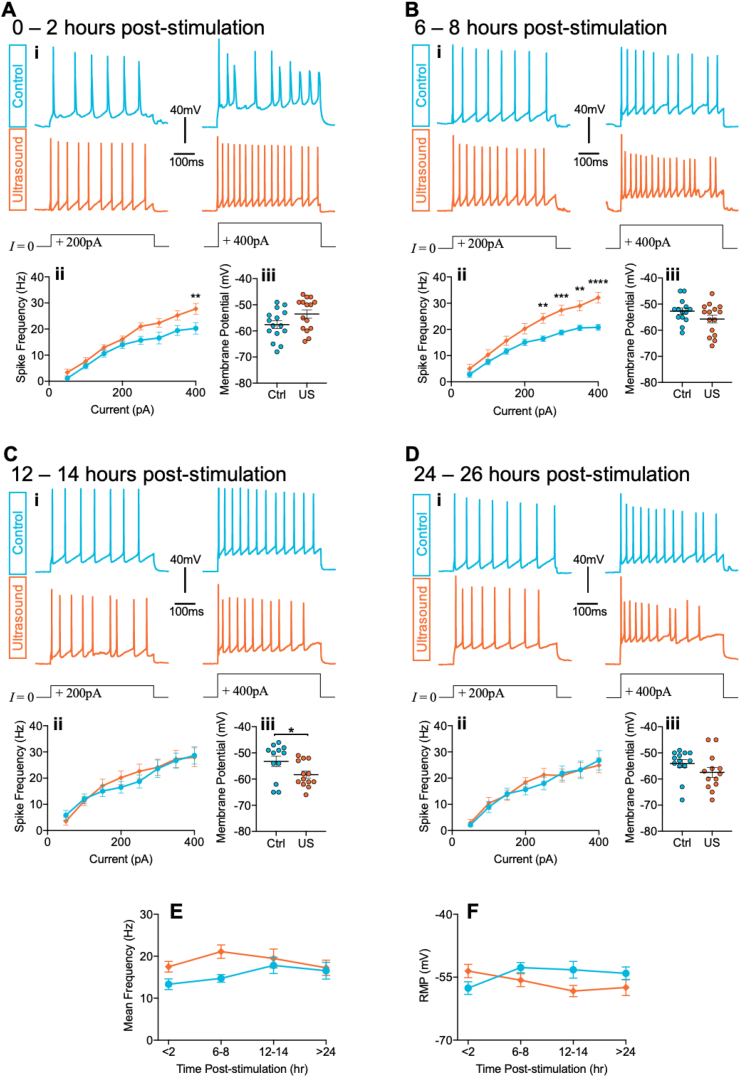
Ultrasound stimulation induces increases in neuronal intrinsic excitability that persists up to 8-h post-stimulation. (A–D) Evoked action potential data at four different time-points (A) 0–2 h, (B) 6–8 h, (C) 12–14 h, and (D) 24–26 h post-stimulation. In each panel (i) Representative voltage responses to 500 ms current pulses of 200 pA (left) or 400 pA (right) from sham (blue) and ultrasound (orange) stimulated cells. (ii) Current evoked spike frequency was significantly increased in ultrasound stimulated neurons at 0–2 h (Aii: effect of ultrasound stimulation: F(1, 28) = 5.77, p = 0.023; effect of current: F(7, 188) = 91.99, p < 0.0001; effect of treatment × current interaction: F(7, 188) = 1.95, p = 0.064), and 6–8 h (Bii: effect of ultrasound stimulation: F(1, 27) = 9.74, p = 0.004; effect of current: F(7, 174) = 187.0, p < 0.0001; effect of treatment × current interaction: F(7, 174) = 9.57, p < 0.0001), but not 12–14 h (Cii: effect of ultrasound stimulation: F(1, 23) = 0.10, p = 0.76; effect of current: F(7, 151) = 41.73, p < 0.0001; effect of treatment × current interaction: F(7, 151) = 0.77, p = 0.61) or 24–26 h (Dii: effect of ultrasound stimulation: F(1, 23) = 0.17, p = 0.69; effect of current: F(7, 149) = 56.24, p < 0.0001; effect of treatment × current interaction: F(7, 149) = 0.58, p = 0.77), analysed by mixed-effects model, between-group (i.e., control vs ultrasound), multiple comparisons testing by Holm-Sidak method. (iii) Resting membrane potential was not significantly different between groups at 0–2 h (Aiii, p = 0.075), 6–8 h (Biii, p = 0.14), or 24–26 h (Diii, p = 0.18). There was a significant hyperpolarisation of ultrasound stimulated neurons at 12–14 h (Ciii, p = 0.044). (E–F) Timecourse summary data displaying significant effect of ultrasound stimulation on (E) mean spike frequency calculated as the mean frequency across all current intensities for a given cell (effect of ultrasound stimulation: F(1, 101) = 7.64, p = 0.007; effect of time: F(3, 101) = 1.49, p = 0.22; effect of treatment × time interaction: F(3, 101) = 1.21, p = 0.31), but not (F) resting membrane potential (RMP), (effect of ultrasound stimulation: F(1, 102) = 2.7, p = 0.10; effect of time: F(3, 102) = 0.46, p = 0.71; effect of treatment × time interaction: F(3, 102) = 3.38, p = 0.021), analysed by 2-way ANOVA. Data is mean ± S.E.M. ∗p < 0.05, ∗∗p < 0.01, ∗∗∗p < 0.001, ∗∗∗∗p < 0.0001. N = (0–2hr) 15 vs. 15; (6–8hr) 14 vs. 15; (12–14hr) 12 vs. 13; (24–26hr) 13 vs. 12 cells (control vs. ultrasound). (For interpretation of the references to colour in this figure legend, the reader is referred to the Web version of this article.)

We first assessed the intrinsic firing properties of cultured cortical rat neurons through whole-cell patch-clamp recording, evoking action potentials in response to somatic current injection. Neurons were first subjected to a 40 s, 200 kHz pulsed ultrasound stimulation or sham-stimulation (see Methods), then transferred to a recording chamber for electrophysiological analysis. The first experimental group consisted of neurons assayed within 2 h of ultrasound stimulation. Step-wise increments in injected current induced progressively increased spike frequency in control, sham-stimulated neurons (Fig. 1Ai, ii; effect of current: *F*(7, 188) = 91.99, *p* < 0.0001). In neurons stimulated with ultrasound, mean spike frequency was significantly increased by 32% above control cell levels (Fig. 1Aii; effect of ultrasound stimulation: *F*(1, 28) = 5.77, *p* = 0.023; effect of treatment × current interaction: *F*(7, 188) = 1.95, *p* = 0.064), indicating enhanced excitability induced by ultrasound stimulation.

**Fig. 2:**
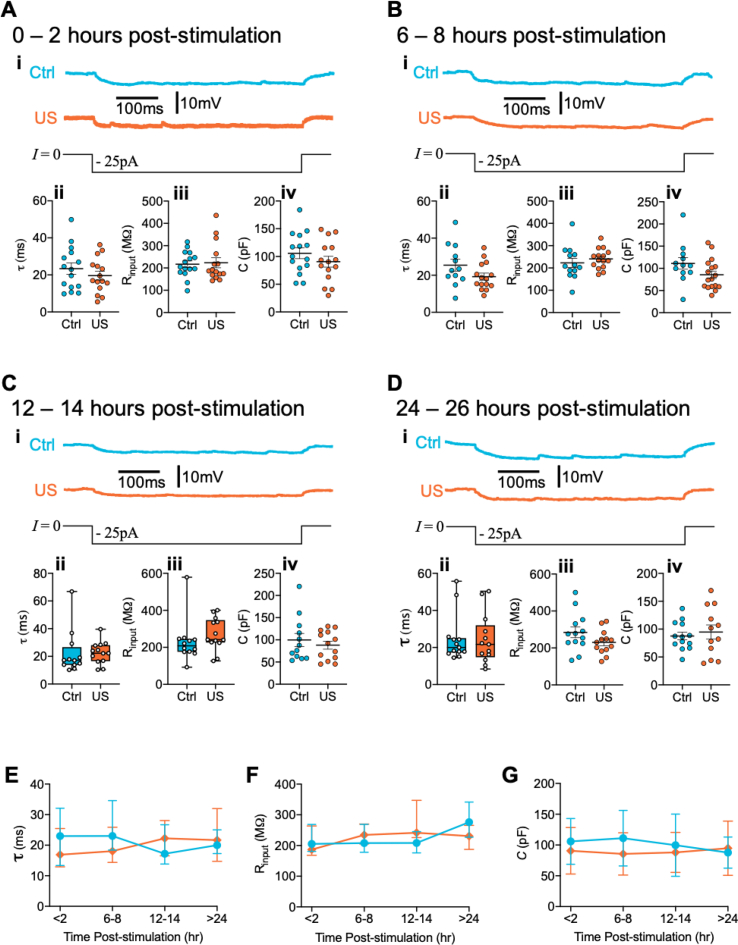
Effect of ultrasound on intrinsic membrane properties. (A-D) Neuronal membrane properties at four time-points (A) 0–2 h, (B) 6–8 h, (C) 12–14 h, and (D) 24–26 h post-stimulation. In each panel (i) Example voltage responses to hyperpolarising current pulse (I = −25 pA) from sham (blue) and ultrasound (orange) stimulated cells, from which the passive membrane properties are derived. (ii) Membrane time-constant (**τ**) derived from a single exponential fit to the voltage trace following the current step, (Aii, p = 0.34; Bii, p = 0.094; Cii, p = 0.35; Dii, P = 0.84). (iii) Input resistance (Rinput) defined as the steady-state voltage deflection divided by the current pulse amplitude, (Aiv, p = 0.81; Biv, p = 0.44; Civ, p = 0.13; Div, p = 0.12). (iv) Membrane capacitance (C) calculated as C = **τ**/Rinput, (Av, p = 0.28; Bv, p = 0.094; Cv, p = 0.50; Dv, p = 0.62, outlier: ultrasound, 397.2 pF). Scatter dot plots line is mean, error is S.E.M., analysed by two-tailed unpaired *t*-test. For (Cii, Ciii, Dii) boxes represent median and inter-quartile range, bars are min and max, analysed by Mann-Whitney test. (E-G) Timecourse summary data for (E) membrane time-constant, (F) input resistance, or (G) capacitance, data is median ± inter-quartile range. N = (0–2hr) 15 vs. 15; (6–8hr) 12 vs. 15; (12–14hr) 12 vs. 13; (24–26hr) 13 vs. 13 cells (control vs. ultrasound).

We next introduced a longer interval, assaying neurons 6–8 h following stimulation (Fig. 1B). There remained a robust 44% increase in mean spike frequency in ultrasound-stimulated compared with sham-stimulated neurons (Fig. 1Bii; effect of ultrasound stimulation: *F*(1, 27) = 9.74, *p* = 0.004; effect of current: *F*(7, 174) = 187.0, *p* < 0.0001; effect of treatment × current interaction: *F*(7, 174) = 9.57, *p* < 0.0001). The significant interaction between ultrasound stimulation and current likely reflects the fact that spike frequency begins to plateau at higher current injections in sham-stimulated neurons, whereas in ultrasound stimulated neurons spike frequency continues to increase approximately linearly. When we introduced a further increase in delay to 12–14 h between stimulation and excitability assay, these effects were eliminated (Fig. 1C; effect of ultrasound stimulation: *F*(1, 23) = 0.10, *p* = 0.76; effect of current: *F*(7, 151) = 41.73, *p* < 0.0001; effect of treatment × current interaction: *F*(7, 151) = 0.77, *p* = 0.61). The effects remained absent at 24–26 h following stimulation (Fig. 1D; effect of ultrasound stimulation: *F*(1, 23) = 0.17, *p* = 0.69; effect of current: *F*(7, 149) = 56.24, *p* < 0.0001; effect of treatment × current interaction: *F*(7, 149) = 0.58, *p* = 0.77). When comparing across the timecourse, we observed a significant effect of ultrasound stimulation on mean spike frequency (Fig. 1F; effect of ultrasound stimulation: *F*(1, 101) = 7.64, *p* = 0.007; effect of time: *F*(3, 101) = 1.49, *p* = 0.22; effect of treatment × time interaction: *F*(3, 101) = 1.21, *p* = 0.31). We found no effect of ultrasound on resting membrane potential coincident with changes to firing frequency (Fig. 1A–D, iii), although there was a marginally significant hyperpolarisation in ultrasound stimulated neurons at 12–14 h (Fig. 1Ciii), and a significant interaction between treatment condition and time (Fig. 1G, effect of ultrasound stimulation: *F*(1, 102) = 2.7*, p* = 0.10; effect of time: *F*(3, 102) = 0.46, *p* = 0.71; effect of treatment × time interaction: *F*(3, 102) = 3.38, *p* = 0.021). This suggests that whilst minor changes to membrane potential may occur due to ultrasound stimulation, this is unlikely to reflect modulation of activity due to a generalised neuronal depolarisation. Moreover, there were no changes to passive membrane properties (Fig. 2) that could otherwise explain the underlying causes of excitability modification. Together, these data indicate that a brief ultrasound stimulus induces modification to neuronal excitability that is sustained for up to 8 h.

**Fig. 3:**
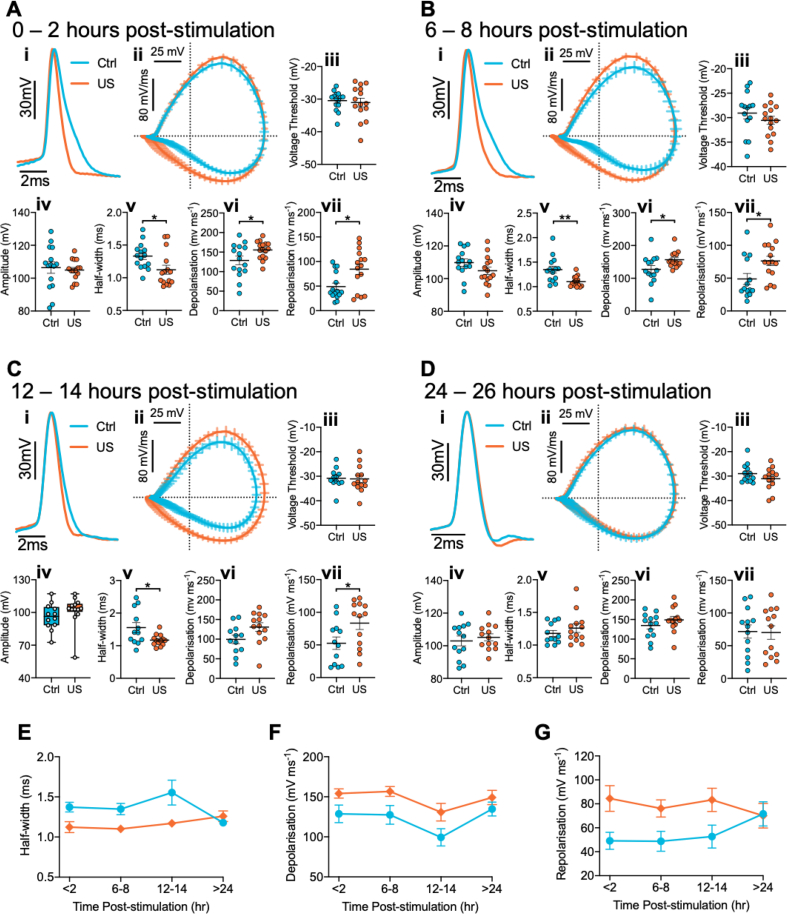
Ultrasound stimulation induces modifications to action potential kinetics that persist up to 14-h post-stimulation. (A–D) Action potential kinetic data at four different time-points (A) 0–2 h, (B) 6–8 h, (C) 12–14 h, and (D) 24–26 h post-stimulation. Data is derived from the first spike in a train evoked by 200 pA injections for each neuron. In each panel (i) Representative action potential waveforms from sham (blue) and ultrasound (orange) stimulated cells, aligned to the voltage threshold and amplitude scaled. (ii) Average phase plots for the first spike evoked by 200 pA injections (data is mean ± S.E.M.). (iii-vii) Summary graphs of (iii) Voltage threshold (Aiii, p = 0.70; Biii, p = 0.30; Ciii, p = 0.88; Diii, p = 0.23), (iv) amplitude (Aiv, p = 0.70; Biv, p = 0.13; Civ, p = 0.14; Div, p = 0.55), (v) half-width (Av, p = 0.013; Bv, p = 0.002; Cv, p = 0.022; Dv, p = 0.33, outlier: ctrl, 2.6 ms), (vi) depolarisation rate (Avi, p = 0.034; Bvi, p = 0.033; Cvi, p = 0.054; Dvi, p = 0.26), and (vii) repolarisation rate (Avii, p = 0.010; Bvii, p = 0.018; Cvii, p = 0.034; Dvii, p = 0.92). Scatter dot plots line is mean, error is S.E.M., analysed by two-tailed unpaired *t*-test. For (Civ) box represents median and inter-quartile range, bars are min and max, analysed by Mann-Whitney test. (E–G) Timecourse summary data displaying significant effect of ultrasound stimulation on (E) half-width (effect of ultrasound stimulation: F(1, 101) = 15.03, p = 0.0002; effect of time: F(3, 101) = 1.597, p = 0.19; effect of treatment × time interaction: F(3, 101) = 3.41, p = 0.02), (F) depolarisation (effect of ultrasound stimulation: F(1, 102) = 14.24, p = 0.0003; effect of time: F(3, 102) = 3.79, p = 0.013; effect of treatment × time interaction: F(3, 102) = 0.31, p = 0.82), and (G) repolarisation (effect of ultrasound stimulation: F(1, 102) = 12.55, p = 0.0006; effect of time: F(3, 102) = 0.295, p = 0.83; effect of treatment × time interaction: F(3, 102) = 1.61, p = 0.19), data is mean ± S.E.M., analysed by 2-way ANOVA. ∗p < 0.05, ∗∗p < 0.01. N = (0–2hr) 15 vs. 15; (6–8hr) 14 vs. 15; (12–14hr) 12 vs. 13; (24–26hr) 13 vs. 13 cells (control vs. ultrasound). (For interpretation of the references to colour in this figure legend, the reader is referred to the Web version of this article.)

To better understand the nature of the sustained effects, we next examined whether excitability modification occurred alongside changes to action potential waveform (Fig. 3A–D, i). We found that the amplitude of the evoked action potentials did not differ between the conditions across the timecourse (Fig. 3A–D, iv). The enhanced excitability could be explained by a reduction in the action potential voltage threshold in ultrasound-stimulated neurons, which would lead to a greater propensity for firing. However, we found no differences in induction threshold between conditions across the timecourse (Fig. 3A–D, iii). Interestingly, there were differences between control and ultrasound-stimulated neurons in half-width, depolarisation rate, and repolarisation rate (Fig. 3A–D, v-vii). These changes were statistically significant across the timecourse (Fig. 3E–G), and similarly eliminated by 24 h following stimulation. These results indicate ultrasound affects action potentials kinetics, concomitant with a sustained increase in propensity for rapid spiking.

**Fig. 4:**
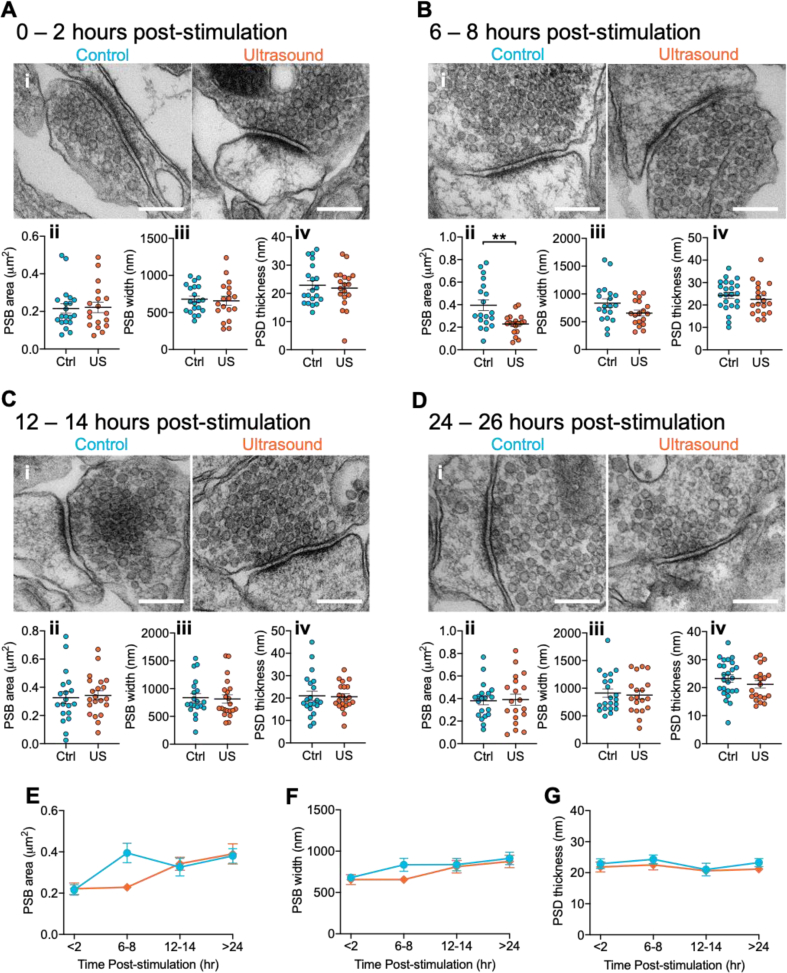
Effect of ultrasound stimulation on synaptic ultrastructure. (A–D) Electron microscopy (EM) analysis of synaptic architecture at four time-points (A) 0–2 h, (B) 6–8 h, (C) 12–14 h, and (D) 24–26 h post-stimulation. In each panel (i) Representative electron micrographs depicting single synapses from sham (blue) and ultrasound (orange) stimulated cells, scale bar = 200 nm. (ii-iv) Summary graphs of (ii) presynaptic bouton (PSB) area (Aii, p = 0.87, outlier: ctrl, 2.0μm2; Bii, p = 0.003; Cii, p = 0.75, outlier: ctrl, 1.3μm2; Dii, p = 0.87, outlier: ctrl 1.1μm2), (iii) PSB width (Aiii, p = 0.75, outlier: ctrl, 1618 nm; Biii, p = 0.071; Ciii, p = 0.82; Diii, p = 0.73), (iv) post-synaptic density (PSD) thickness (Aiv, p = 0.65, outlier: ctrl, 67 nm; Biv, p = 0.41; Civ, p = 0.86; Div, p = 0.27), Data is mean ± S.E.M., analysed by two-tailed unpaired *t*-test. (E–G) Timecourse summary data displaying for (E) PSB area (effect of ultrasound: F(1, 146) = 1.697, p = 0.19; effect of time: F(3, 146) = 7.37, p = 0.0001; effect of treatment × time interaction: F(3, 146) = 2.922, p = 0.036), (F) PSB width (effect of ultrasound stimulation: F(1, 148) = 1.82, p = 0.18; effect of time: F(3, 148) = 4.02, p = 0.009; effect of treatment × time interaction: F(3, 148) = 0.582, p = 0.63), and (G) PSD thickness (effect of ultrasound stimulation: F(1, 160) = 1.489, p = 0.22; effect of time: F(3, 160) = 0.967, p = 0.41; effect of treatment × time interaction: F(3, 160) = 0.123, p = 0.95), data is mean ± S.E.M., analysed by 2-way ANOVA. ∗∗p < 0.01, (A-Dii, iii, E & F) N = (0–2hr) 21 vs. 18; (6–8hr) 19 vs. 18; (12–14hr) 20 vs. 21; (24–26hr) 21 vs. 19. (A-Div, G) N = (0–2hr) 20 vs. 19; (6–8hr) 23 vs. 19; (12–14hr) 21 vs. 21; (24–26hr) 25 vs. 20 synapses (control vs. ultrasound). (For interpretation of the references to colour in this figure legend, the reader is referred to the Web version of this article.)

The acoustic radiation force of ultrasound displaces cell membranes [18]. Given that perturbations to morphology can profoundly affect neuronal excitability [19], we wondered whether the effects of ultrasound stimulation that we had observed were associated with changes to neuronal structure. For instance, enlargement of synaptic boutons has been observed in response to high frequency action potential firing [20]. Furthermore, previous evidence suggests that ultrasound affects synaptic regions [12,17]. We therefore decided to examine possible ultrastructural change at pre- and post-synaptic boutons. We prepared fixed samples of control sham- or ultrasound-stimulated neurons and performed electron microscopy imaging (Fig. 4A–D, i). We found no difference in pre-synaptic bouton diameter or post-synaptic density (PSD) thickness across any of the experimental conditions (Fig. 4A–D, iii, iv; summarised data G and F). We did observe a significant effect on pre-synaptic bouton area at 6–8 h but at no other time-point (Fig. 4A-Dii), and this was not accompanied by a significant effect of ultrasound across the timecourse (Fig. 4E). The absence of any changes to synaptic ultrastructure at 0–2 h, and only a marginal effect on bouton area at 6–8 h, suggests that while brief ultrasound stimulation causes sustained modification to neuronal excitability this is not associated with major changes to synaptic ultrastructure.

## 3 Discussion

Our observation of a sustained – but not indefinite - change in neuronal excitability in response to ultrasound stimulation is suggestive of a plastic-like modification to the firing properties of stimulated cells. Previous evidence suggests that transient, patterned neuronal stimulation can rapidly modify intrinsic excitability [21]. These effects share parallels with synaptic plasticity, the widely-studied set of cellular mechanisms responsible for activity-dependent modification of synaptic strength. Indeed, activity-dependent modification of synaptic and excitability properties likely co-occur [22]. The timecourse of the excitability effects we observed – peaking at 6–8 h and returning to control levels by 12 h – is also consistent with a transient, short-term plasticity of excitability [23]. Interestingly, we do not find major changes to synaptic ultrastructure coincident with enhanced neuronal excitability in response to ultrasound stimulation. The size of the pre-synaptic active zone has been demonstrated to be positively correlated with neurotransmitter release probability [24]. Our observation of a reduction in pre-synaptic bouton area at the single 6–8 h timepoint may therefore be associated with decreased release probability at this timepoint, though further investigation will be necessary to fully characterise this.

Whilst our experiments have employed juvenile neurons, similar observations have been reported in adult brains [7,8]. Indeed, in our study, we have used a 200 kHz ultrasound wave, delivered in 100 ms pulses at 100 ms intervals, for 40 s, equating to a pulsing frequency of 5 Hz. These parameters are similar to those of recent *in vivo* studies in adult macaques reporting target specific, bidirectional changes to functional connectivity that persist up to 2 h following stimulation [7,8], thus the findings here may reflect some of the underlying changes to neuronal physiology underpinning the *in vivo* observations.

Importantly, these studies did not collect data beyond 2 h following stimulation so the longer-term consequences of US in these reports are unknown. Conversely, ultrasound-mediated suppression of somatosensory evoked potentials peaked 5 min post-stimulation and returned to baseline within 20 min [25]. Similarly, ultrasound-mediated modulation of oculomotor behaviour was reversed within 18–31 min of stimulation [26]. These differences in temporal characteristics may be due to differences in stimulation parameters or brain regions targeted, and the fact that *in vivo* target regions are stimulated in a focally restricted manner, whereas our entire culture is stimulated simultaneously. Furthermore, Legon and colleagues report a 3.7–4.1 fold decrease in focal point intensity at 500 kHz when transmitting through the skull compared to transmission in free space [10]. Additionally, the glass coverslip we use acts as an acoustically hard reflector and hence changes the local intensity in the vicinity of the cells relative to *in vivo*. In the *in vivo* case the radiation force arises mainly from absorption, whereas *in vitro* (on the coverslip) there is an additional gradient forcing effect. At present the relative importance of these contributions is not known.

The mechanisms by which neurons are sensitive to this high-frequency ultrasound stimulation are still to be determined, though there are an array of potential candidates [6]. Voltage-gated ion channels are the principal components of action potential generation and therefore excitability. Indeed, altered neuronal excitability is directly linked with the modulation of voltage-gated ion channels [21,27]. Voltage-gated Na^+^ channels (Na_v_) and K^+^ channels (K_v_) play key roles in action potential generation. Importantly, changes in the function or expression of Na_v_ and K_v_ impact neuronal excitability [21]. Here we find changes to the waveform of action potentials in ultrasound-stimulated neurons (Fig. 3), specifically the depolarisation and repolarisation rates, which are largely governed by Na_v_ and K_v_ channels. Interestingly, previous studies have shown that ultrasound modulates K_v_ [13] and Na_v_ [14] channel kinetics. What remains to be understood, however, is how the brief ultrasound stimulation results in apparent sustained change in the function of Na_v_ and K_v_ channels. Various kinases have been demonstrated to regulate Na_v_ and K_v_ channel function leading to excitability modifications [28,29]. One possibility, therefore, is that ultrasound induces cellular Ca^2+^ flux [12], initiating Ca^2+^-sensitive signalling and kinase activation to modulate channel function. Understanding the intracellular signalling cascades regulated by ultrasound, therefore, will be an important next step in characterising its mode of action.

## 4 Conclusion

Ultrasound offers significant advantages over other non-invasive neuromodulatory tools, principal of which is its spatiotemporal precision. Our findings suggest that beyond stimulating specific neuronal nuclei to induce immediate responses, ultrasound can additionally, and perhaps most beneficially, be used as a conditioning tool. In this regard, it could be used in circumstances where a general sustained enhancement (or inhibition) of activity is desirable. Beyond experimental applications, such approaches could be greatly beneficial in disease therapy, where enhancing activity (such as dopaminergic cell stimulation in Parkinson’s disease), or reducing activity (such as in epilepsy), could have significant therapeutic impact.

## 5 Methods

**Primary rat cortical neuronal cultures.** Cortical neurons were cultured from post-natal day 0 male Wistar rats, in accordance with established methodology [30]. Briefly, following Schedule 1 killing of the animal, the brain is removed and transferred to HABG media (HibernateA, B-27 Supplement and Glutamax) before dissection. Cortical tissue is then pulled apart into approximately 2 mm^3^ sections, then digested with Trypsin-EDTA. Neurons are isolated using a Density Gradient Medium (OptiPrep), and finally plated onto 15 mm diameter glass poly-d-lysine-coated coverslips at a density of 3 × 10^4^ per cm^2^ in NeurobasalA media (NeurobasalA, B-27 Supplement, Glutamax and Gentamicin). Cultured cells were incubator-stored at 20% O_2_, 5% CO_2_, 37 °C.

**Electrophysiology.** Conventional whole-cell patch clamp recording was used in accordance with our established protocols [31]. Briefly, recordings were made from primary rat cortical cultured neurons at DIV 21–30. Coverslips with plated neurons were placed in a recording chamber submerged in HEPES-buffered saline (HBS) containing: 119 mM NaCl, 5 mM KCl, 25 mM HEPES, 33 mM glucose, 2 mM CaCl_2_, 2 mM MgCl_2_, 1 μM glycine, 100 μM picrotoxin, pH 7.4 adjusted with NaOH, flowing at 2 ml/min. Glass microelectrodes were pulled by a micropipette puller P1000 (Sutter Instrument, Novato, California, USA) with resistances ranging from 4 to 8 MΩ after filling with internal solution containing: 135 mM K-gluconate, 10 mM HEPES, 0.5 mM EGTA, 2 mM Mg-ATP, 0.3 mM Na-GTP, 8 mM NaCl, pH 7.2 adjusted with KOH, osmolarity 285 mOsm. Recordings were made using an Axon Axopatch 200 B Microelectrode Amplifier (Axon Instruments, Molecular Devices, California, USA). Evoked action potentials were recorded in response to sequential stepwise current injections ranging from −50 pA to +400 pA. Passive membrane properties were derived from recorded voltage responses to −25pA current injections. Amplitude, frequency and kinetics of events, in addition to passive membrane properties, series resistance and input resistance were monitored online and reanalysed offline, using the WinLTP [32] and Clampfit (Molecular Devices, USA) software.

**Ultrasound stimulation.** Neurons were transferred to a chamber and submerged in HBS. A 200 kHz ultrasound transducer (MCUSD19A200B11RS, Farnell, UK) powered by a signal generator (AFG3022B, Tektronix, USA) amplified by a radio frequency amplifier (25A250, Amplifier Research, USA), was used to generate the ultrasound pulse. The transducer was excited with a 200 kHz sinusoidal wave of amplitude 50 V peak-to-peak, delivered in 100 ms pulses at 100 ms intervals, for 40 s, equating to a modulation frequency of 5 Hz. Control, sham-stimulation involved the same procedure of cells being placed in the stimulation chamber for equal time, with all equipment powered on, but no excitation signal being generated. Following stimulation, cells were transferred to a recording chamber for electrophysiological analysis.

**Electron Microscopy.** Following stimulation coverslips were fixed at the relevant timepoint with 2.5% glutaraldehyde in 0.1 M sodium cacodylate buffer. Samples were postfixed with 1% osmium tetroxide, washed, and then stained *en bloc* in 3% aqueous uranyl acetate. Coverslips were dehydrated using an ethanol series and embedded in EPON812 resin. The resulting blocks were sectioned with a Leica EM UC7 ultramicrotome (Leica Microsystems GmbH, Wetzlar, Germany) at 70 nm. Sections were poststained with 3% aqueous uranyl acetate and lead citrate. Imaging was carried out with a FEI Tecnai 12 120 kV BioTwin Spirit TEM with tungsten filament and Ceta 4 k x 4 k CCD camera (Thermo Fisher Scientific, Waltham, USA). Images were captured at a magnification of 23000x.

**Image Analysis.** TEM images were analysed using FIJI/ImageJ [33,34]. Bouton area was calculated using the polygon tool to trace around the presynaptic region. Bouton width was determined by measuring a line parallel with the electron dense PSD at the widest point of the polygon mentioned above. PSD thickness was calculated by dividing the area of the PSD including the electron dense scaffold by the length of the postsynaptic membrane as previously described [35].

**Data analysis and statistics.** After initial analyses in respective software (Clampfit/FIJI), resulting data was transported into GraphPad Prism (macOS v8.4.3, GraphPad Software, San Diego, California, USA), for statistical analysis and graphical representation. Data was tested for normality by D’Agostino and Pearson K2 test (*p* < 0.01). In some instances, normality was restored by removal of a single highly significant outlier (ROUT test, FDR < 0.1%). These instances have been reported in the relevant figure legends, along with the value of the outlier. Where data was normally distributed, it has been presented as mean ± S.E.M. (i.e., scatter dot plots), where it was not, data has been presented as median with inter-quartile range (i.e., box-and-whisker plots). Non-normal data was analysed by Mann-Whitney *U* test. Normally distributed data was analysed by either two tailed unpaired *t*-test, 2-way analysis of variance (ANOVA), or mixed-effects model as indicated in the figure legends. Holm-Sidak method was used for between group (i.e., control vs ultrasound) multiple comparisons testing. The data reported in this study are available from the corresponding author upon reasonable request.

## Credit author statement

Benjamin Clennell: Conceptualization; Data curation; Formal analysis; Investigation; Methodology; Roles/Writing - original draft, Writing – original draft; Writing – review & editing. Tom G.J. Steward: Conceptualization; Formal analysis; Investigation; Methodology;, Writing – original draft Roles/Writing - original draft; Writing – review & editing. Meg Elley: Investigation; Methodology. Eunju Shin: Conceptualization; Funding acquisition; Supervision. Miles Weston: Conceptualization; Funding acquisition; Methodology; Supervision. Bruce W. Drinkwater: Conceptualization; Funding acquisition; Methodology; Supervision; Writing – review & editing. Daniel J. Whitcomb: Conceptualization; Data curation; Funding acquisition; Methodology; Project administration; Resources; Supervision; Roles/Writing - original draft, Writing – original draft; Writing – review & editing.

## Declaration of competing interest

The authors declare that they have no known competing financial interests or personal relationships that could have appeared to influence the work reported in this paper.
